# Mr. Rogerson's Jacksonian Prize—Dissertation on Inflammation of the Membranes

**Published:** 1834-04-01

**Authors:** 


A TREATISE ON INFLAMMATIONS, &c. &c. BEING AN EXTENSION OF A DISSERTATION ON INFLAMMATION OF THE MEMBRANES, TO WHICH THE JACKSONIAN PRIZE FOR THE YEAR 1828, WAS AWARDED BY THE ROYAL COLLEGE OF SURGEONS IN LONDON. By *George Rogerson*, Surgeon, of Liverpool. 8vo. pp. 459. Vol. I. London, 1832.

IT requires no small degree of ardour and of courage to read or to write, at this time of day, a systematic treatise upon inflammation. In the first place, it is a subject which has occupied the minds and pens of many eminent men, with only an indifferent degree of success; and, in the next, the taste of the times, is by no means favourable to general dissertations. Nosologies and systems are rather things that were, than growths of the present age, and men evince a disposition to study facts in preference to speculative reasonings. The writings which now attract most attention are those which record facts, or are little more than inductions from them, works, in short, of a clinical character. We need not mention particular examples, as they cannot fail to present themselves to the recollection of all who are conversant with recent medical literature.

We do not affirm that this taste should be exclusively indulged in, although its effects are, on the whole, most salutary. It is true that men of great talent or ingenuity, are discouraged from exercising those qualities on speculative points, and we are not likely to have other Cullens, Boerhaaves, or Browns. The day of medical, like that of parliamentary *leaders* is past, and although the existing order of things is less calculated for the vivid displays of genius, it is better adapted for business—though it may strip talent of some of its glitter, it will probably tend to render it more steady and more permanently useful.

Works, the object of which is the enunciation and elucidation of general principles, should not be rashly attempted by ordinary men. It requires a masterly and analytic mind to succeed in such an enterprize. General principles in medical science, should be as purely inductive as possible, and when we consider the immense number of facts that have been accumulated and present themselves to daily notice, the many modifying circumstances that affect them, and the varieties, nay, contradictions, that they display, we feel no surprise that hitherto the majority of writers have failed in their ambitious endeavours at systematic analysis and combination. We fear that the time for these comprehensive essays is not yet come, that individual facts must be farther studied, limited inductions more clearly established, before there will be a sound and at the same time ample foundation for their support. The laws of inflammation, for instance, are, when properly considered, merely conventional expressions of the phenomena of inflammation, mere modes of stating in abbreviated forms the frequency of such and such occurrences. A correct exposition of the laws, presupposes therefore an accurate knowledge of the facts. But are all the facts yet known to us? It is only within these last few years that we have received a true account of the character and progress of phlebitis—of the secondary inflammations and deposites—of the differences displayed by inflammation in the different tissues of the joints.* It is evident that a man living at the period when Hunter did, a period antecedent to that in which these facts were known, could not possibly construct an accurate treatise on inflammation, when such a treatise should comprise circumstances of which he was absolutely ignorant. We are thus thrown back on observation, we are taught to generalize cautiously and slowly, and in this way the materials are laboriously accumulating for wider and more universal inductions.

We have been induced to make these few remarks by a hurried glance at the volume before us. We fear our author has been unwise in embarking on so bold a venture, we fear he has in some measure over-estimated his strength, and exposed himself to criticism instead of commanding conviction. We are bound to shew that we have not judged harshly, nor condemned unjustly. We will take at hazard the fourth section of the volume, the subject of which is the classification of inflammation. It opens in the following manner.

"All parts of the body are subject to attacks of inflammation, and the disorder consists of several well-marked varieties or species, affecting membranes and organs in a different manner. All maladies, of any degree of seriousness, appertain to one or other of these species of inflammation; and the diseases of all the structures of the body, of every kind, are caused by them: but the great multiplicity and variety of symptoms observed in one species of inflammation are owing solely to the difference of the anatomy and physiology of the respective tissues and organs. Pathology, though comprising the whole catalogue of the suffering ills of living organization, is necessarily confined to general maladies, few in number: but its application to the different structures of the body will cause it to assume forms, or give signs, as numerous and variable as the structures composing that living body differ in organization. The present complicated nosologies are, therefore, not only perfectly useless, but injurious, since the cause of the pathological states of structure is clearly attributable to one variety or other of inflammation; and what these varieties are, we will now proceed to examine." 77.

The critic will discover in this brief passage several assumptions, and much obscurity. It is surely an assumption that "all maladies of any degree of seriousness appertain to one or other of the species of inflammation, and that the diseases of all the structures of the body, of every kind, are caused by them." It has never been proved that fungus hæmatodes is a consequence of inflammation—nor that scirrhus is such a consequence—nor epilepsy—nor convulsions—nor tetanus—nor hydrophobia—nor passive dilatation of the heart, nor many other serious maladies to which we might readily refer. It is true that it is supposed by some that inflammation of some peculiar kind *may* give rise to some of these affections, for instance, to the morbid growths; but this is a supposition, or at best a moot point, and certainly not sufficiently established to admit of a confident enunciation. How the little modicum of reasoning with which we are presented, or how, indeed, in the present state of science any quantum of reasoning whatever

* On some of these subjects modern investigation has still much to do. Phlebitis and the purulent deposites are neither well described nor understood.—*Ed.*

can warrant a philosophic writer in asserting, that the cause of all morbid alterations of structure is attributable to some variety of inflammation, we really cannot conceive. It is a palpable assumption.

Mr. Rogerson criticizes, and we think successfully, Mr. Hunter's divisions of inflammation into the adhesive, suppurative, &c. Adhesion, suppuration, are effects of inflammation, not varieties of the process itself, and we sometimes observe at the same time, and in the same inflammation, adhesions, suppuration, sloughing. Mr. Rogerson also notices the opinion which has been advanced, that the varieties of inflammation are owing to the difference of texture. To this theory Mr. Hunter well objected, that inflammation of one character will, at the same instant, attack many tissues; thus, after an amputation of a limb, inflammation will occur, and end in adhesion or in suppuration, this state being observed in the various tissues of skin, cellular membrane, muscle, tendon, bone. Inflammation is greatly modified by tissue, and certain textures display a preference for peculiar forms and terminations of inflammation; but its varieties cannot be considered as merely the result of the influence of tissue.

After freely commenting on other divisions of inflammation, Mr. Rogerson proposes his own, which is this. He arranges the different species of inflammations into four classes, "in which they are considered according to the general state of the affected part during its inflammatory stage." Those classes are as follows:—

> "First. Inflammations which are limited to a certain extent on structure.
>
> Second. Inflammations whose dispositions are to spread.
>
> Third. Inflammations which, during the inflammatory stage, disorganize structure, converting it into a nature *sui generis*.
>
> Fourth. Inflammations which arise from some morbific or poisonous matter on structure." 89.

In short, the varieties of inflammation may be termed the limited or limiting—the spreading—the disorganizing—and the poisonous.

> "Each species has a partiality for some particular membrane, or parts of it, in preference to any other; thus common inflammation is seated more particularly in the cellular membrane; erysipelas in the skin; carcinoma, and scrofula, in the glandular parts of a membrane; and poisons affect the structures, as if they were allotted to them." 89.

Objections to this scheme very readily present themselves. The inflammation which follows the reception of a morbid poison may display the first or the second character—may be strictly limited, or rapidly diffused. We need not reiterate the reasons which induce us to reject inflammation as the origin of many morbid changes of structure.

Perhaps the varieties of inflammation may be analytically reduced to two —the limited and the diffuse. The former evinces a marked disposition to the effusion of coagulated lymph at its periphery, and to limitation by this salutary cordon; the latter runs along a tissue, producing disorganization in one case, and comparatively little alteration in another. Striking instances of this kind of inflammation are presented in erysipelas, and diffuse inflammation of the subcutaneous or intermuscular cellular membrane.

We might possibly be tempted to add to these two species a third—the inflammation dependent on specific poisons, as lues, small-pox, vaccinia, inoculation from dissection-wound, and so on. But, even here, a close examination serves rather to detect distinctions in the phenomena accompanying the inflammation, than in any essential features of the latter. But we will not pursue this seductive subject.

When two or more membranes are inflamed, Mr. Rogerson terms the inflammation compound. We notice this for a reason that will shortly be apparent. Mr. Rogerson proceeds to mention, that the cellular structure and the skin are frequently affected at the same time, and that this affection has been improperly denominated phlegmonous erysipelas—erysipelas phlegmonodes, &c. We will let Mr. R. declare his own opinions.

"The compound erysipelas most frequently takes place between some membrane and its adjacent cellular texture. It is often observed that the cutaneous and subcellular membrane are attacked, when it has erroneously and improperly been named 'erysipelas phlegmonoides,' 'phlegmonous erysipelas,' &c. But this name is erroneously and improperly given, because it serves to convey a wrong idea of the nature of the disease. The title being compounded of phlegmon and erysipelas, certainly expresses that this disorder is a compound of the two varieties of inflammation, which is not the case. It is one variety of inflammation, but seated in two separate kinds of membranes, which must create some variations in the symptoms from those of the simple inflammation of either of them, as well as in the course of the disorder. The term, then, is highly objectionable; for, from the construction which its derivatives admit, it would imply that two different and distinct species of inflammation co-existed in the same part at the same time, which Mr. Hunter has proved to be impossible; and it is to the establishment of this golden rule, that medicine is indebted for its great pathological and nosological simplicity. It is owing to a negligence of this rule, too, that the term was applied, from a fancied resemblance to two maladies; for men are naturally prone, in their descriptions of new facts and discoveries, to form similes from, or draw comparisons between, objects already known. This nosology seems to be tinctured with the opinions propagated by Dr. C. Smyth, for here phlegmon might be applied to the inflammatory malady of the cellular membrane, and erysipelas to that of the skin. This combined term should, however, be discontinued; and surely it would be much better to substitute a name expressive of the real nature of the disorder, as the compound erysipelas of the cutaneous and cellular membranes. By so doing, we learn that the inflammation is erysipelas, and that it is existing in two neighbouring membranes at the same times." 98.

We agree with our author in deeming the term "phlegmonous erysipelas" a bad one. But we do not altogether approve of his substitute. If many of these cases are observed with care, they will not appear to be erysipelas at all. The disease very frequently commences as diffuse inflammation of the subcutaneous cellular membrane, and the blush upon the skin is consecutive in regard to time, and inferior in extent. In the limb of a person who has received a compound fracture, or some lacerated wound, there is frequently an opportunity of witnessing distinctly the commencement of the disease in the cellular texture—the subsequent invasion of the skin—the absence of the defined erysipelatous border to the redness of the latter—the extent of effusion, of suppuration, and of sloughing in the cellular tissue, prior to ulceration or to sloughing of the skin. We have never yet seen a *very* good account of this affection. Mr. Lawrence's is certainly imperfect, and in some degree erroneous.

There is a subject to which we could have willingly alluded, one of the greatest practical importance, and of no mean physiological interest. It is the consideration of the fevers of inflammation.

Our space will not permit us to enter on this interesting subject. Adequate justice could not be done to it in a hurried notice of a few pages. In our next we shall probably return to it, and endeavour to lay before our readers some curious and important facts.

